# Editorial: Searching for the Boundaries of Microbial Speciation in a Rapidly Evolving World

**DOI:** 10.3389/fmicb.2021.808595

**Published:** 2021-12-23

**Authors:** Eric Daniel Becraft, Andrés Moya

**Affiliations:** ^1^Department of Biology, University of North Alabama, Florence, AL, United States; ^2^Institute for Integrative Systems Biology, University of Valencia and Spanish Research Council (CSIC), Valencia, Spain; ^3^Foundation for the Promotion of Sanitary and Biomedical Research of Valencian Community (FISABIO), Valencia, Spain; ^4^CIBER in Epidemiology and Public Health (CIBEResp), Madrid, Spain

**Keywords:** microbial diversity, microbial evolution, speciation, ecotypes, population genetics

## Introduction

Whether bacteria and archaea form species-like populations has been a long-standing debate. In contrast to eukaryotic organisms, horizontal gene transfer in microorganisms can make observing vertical lineages of descent difficult. Microorganisms evolve very fast, most are difficult to culture, and the number of cells in a population and community can often be >10^9^ cells/ml, all making studying the process of evolution and speciation difficult. There is evidence to support the idea that bacteria and archaea form cohesive sequence clusters (Jain et al., 2018; Cohan, 2019), that they form ecological species (Becraft et al., 2015; Cohan, 2017), that the rate of homologous recombination can be used as the defining species barrier (Vos and Didelot, 2009; Cadillo-Quiroz et al., 2012; Bobay and Ochman, 2017), and that microbial species simply do not exist due to speed of evolution and a high rate of horizontal gene transfer (Doolittle and Papke, 2006; Papke et al., 2007). Often the system or organisms analyzed effect how researchers subsequently define species, though until about 10 years ago sequencing technologies and techniques were not adequate to answer many of these questions *in situ*. The rapid increase in sequencing technologies, such as metagenomics and single cell genomics, is allowing researchers to once again approach this topic with higher genetic resolution.

## Summary of Contributed Publications

While there are varying mechanisms of genetic change and speeds of evolution, the forces acting upon genetic diversity are consistent across the tree of life. In this Research Topic, we asked researchers to share studies on a multitude of systems and taxonomic groups to observe if patterns emerge in regards to microbial species and speciation. This should lead to similarities and principles that can be applied across the tree of life and in more complex ecosystems critical to our planet, such as soils, ocean water, and the human microbiome. By understanding microbial speciation and the fundamental units within an ecosystem we can better understand how ecosystems adapt to environmental change, contribute to geochemical cycling, or cause disease.

In the article entitled “*The Origin of Niches and Species in the Bacterial World*,” Baquero et al. examined the complexity of the niche and examine a multitude of ways that bacteria and archaea might speciate and exploit those niches as populations diversify over time. In the microbial world, the concept of niche implies that a microbial organism is present or was present in the recent past (empty niche) at a particular multidimensional environmental space. However, the microbial population interacts with the niche, creating sub-niches that are colonized by variant sub-populations of bacteria. Such a diversification process is compensated by the genetic interactions of sub-niche bacterial variants, leading to an evolved population able to fully exploit the expanded niche. Optimization occurs when the full carrying capacity of the niche is reached, and such perfect niche-bacteria trade-off facilitates specialization and eventually genetic isolation and speciation. In summary, the origin of species and the origin of niches are entirely intertwined.

The article entitled “*Insights on the Evolutionary Genomics of the Blautia Genus: Potential New Species and Genetic Content Among Lineages*,” Maturana and Cárdenas used a pangenomic analysis combined with a whole-genome approach to reevaluate a previously known taxonomic group, which both discovered new species and reassigned others. Analysis of hundreds of publicly available *Blautia* strain genomes found 17 previously undescribed species and helped to reconstruct important gene gain/loss events during the diversification of the *Blautia* genus, shedding light about how this genus specialized to live in the gastrointestinal tract.

The article entitled “*Comparative Genomic Insights into the Taxonomic Classification, Diversity, and Secondary Metabolic Potentials of Kitasatospora, a Genus Closely Related to Streptomyces*,” Li et al. used comprehensive phylogenomics and comparative genomics analyses to examine genomic differences among lineages within the genus Kitasatospora. It revealed genomic differences between the closely related genera Streptomyces and Streptacidiphilus, supporting Kitasatospora as a separate genus within the family Streptomycetaceae. Comprehensive annotation also uncovered unexpected potentials of secondary metabolism in Kitasatospora, which carries more and longer secondary metabolite biosynthetic gene clusters than the well-known antibiotic producer Streptomyces.

The article entitled “*Phylogenetic Distribution of Polysaccharide-Degrading Enzymes in Marine Bacteria*,” Sun et al. used large-scale genomics to annotate polysaccharide-degrading enzymes in marine bacterial genomes based on a manually curated enzyme sequence database. The authors found extended phylogenetic distributions for most enzymes analyzed. Interestingly, there was substantial intra-clade diversity in the coding potential of polysaccharide-degrading enzymes, indicating diversification in the polysaccharide-utilization abilities and thus niche differentiation. The study suggested that the polysaccharide-degrading enzymes could be used as molecular markers for marine bacterial niche adaptation and speciation.

The article entitled “*Host- and Species-Dependent Quasispecies Divergence of Severe Acute Respiratory Syndrome Coronavirus-2 in Non-human Primate Models*”, Hwang et al. examined newly emerging SARS-CoV-2 variants that have been reported to cause higher morbidity and mortality than previous viruses despite enforcement of strict biosecurity and vaccination policies. Based on single nucleotide polymorphism analysis, the high intra-host, inter-host and inter-species genomic variability of SARS-CoV-2 following a short replication period were verified in Cynomolgus monkeys and *Rhesus macaques* infected with identical SARS-CoV-2. These results are valuable not only for understanding SARS-CoV-2 variant evolution in hosts, but also for development of new diagnostics, vaccines, and therapeutics targeting SARS-CoV-2.

The article entitled “*Comparative genomics of novel Agrobacterium G3 strains isolated from the International Space Station and description of Agrobacterium tomkonis sp. nov*.,” Singh et al. used a polyphasic approach to characterize the phenotypic and genotypic synapomorphies of a novel *Agrobacterium tomkonis*, showing it is a bacterial species, well-separated from previously named *Agrobacterium* species. *A. tomkonis* are diverse and have been isolated from hospital, plant rhizosphere soil, and the International Space Station surfaces. *A. tomkonis* specific gene functions notably relate to surface adhesion and could be involved in the ecological specificity of *A. tomkonis*, as suggested by their varied source of isolation. The ability to colonize nutrient-poor substrates, unusual for most agrobacteria, resonate with the finding that *A. tomkonis* genomes specifically carry genes involved in mediating attachment to surfaces, including production of putative adhesins and biofilm production. This suggests a particular ability of *A. tomkonis* to colonize different habitats that are even poorer in nutrients and harsher than soil, thus possibly escaping competition with other agrobacteria that are better at growing in richer environments, such as plant rhizospheres.

## Do Species Matter in Microbial Communities?

Using modern sequencing technologies and bioinformatic techniques, researchers observed patterns of microbial diversity across multiple taxa and in numerous distinct systems. Observations were made of microbial populations evolving to unique ecological niches, regardless of taxa or environment. While horizontal gene transfer can allow for niche adaptation and act as cohesive force among populations, the observed populations appeared to have diversified though selection to unique, and sometimes subtle, ecological niches. Continued research combined with improving technology will allow researchers to determine if a common species definition can ultimately be applied across all bacterial and archaeal lineages.

## Author Contributions

All authors listed have made a substantial, direct, and intellectual contribution to the work and approved it for publication.

## Conflict of Interest

The authors declare that the research was conducted in the absence of any commercial or financial relationships that could be construed as a potential conflict of interest.

## Publisher's Note

All claims expressed in this article are solely those of the authors and do not necessarily represent those of their affiliated organizations, or those of the publisher, the editors and the reviewers. Any product that may be evaluated in this article, or claim that may be made by its manufacturer, is not guaranteed or endorsed by the publisher.
